# *TGFBR3 *variation is not a common cause of Marfan-like syndrome and Loeys-Dietz-like syndrome

**DOI:** 10.1186/1477-5751-11-9

**Published:** 2012-02-02

**Authors:** Krishna K Singh, Joerg Schmidtke, Britta Keyser, Mine Arslan-Kirchner

**Affiliations:** 1 Institute of Human Genetics, Hannover Medical School, Hannover, Germany; 2 Division of Cardiac Surgery, St. Michael's Hospital, Toronto, Canada

**Keywords:** MFS, LDS, *TGFBR3*, *variants*

## Abstract

Marfan syndrome (MFS) is caused by mutations in the fibrillin-1 (*FBN1*) gene, and mutations in *FBN1 *are known to be responsible for over 90% of all MFS cases. Locus heterogeneity has also been reported and confirmed, with mutations in the receptor genes *TGFBR1 *and *TGFBR2 *identified in association with MFS-related phenotypes. It is now known that dysregulation of TGF-ß signaling is involved in MFS pathogenesis. To test the hypothesis that dysregulation of TGFBR3-associated TGF-ß signaling is implicated in MFS or related phenotype pathogenesis, we selected a cohort of 49 patients, fulfilling or nearly fulfilling the diagnostic criteria for MFS. The patients were known not to carry a mutation in the *FBN1 *gene (including three 5' upstream alternatively spliced exons), the *TGFBR1 *and *TGFBR2 *genes. Mutation screening for the *TGFBR3 *gene in these patients and in controls led to the identification of a total of ten exonic (one novel), four intronic (one novel) and one 3'UTR variant in the *TGFBR3 *gene. Our data suggest that variations in *TGFBR3 *gene appear not to be associated with MFS or related phenotype.

## Background

Marfan syndrome (MFS; MIM# 154700) is an autosomal-dominant disorder of connective tissue with major manifestations in the skeletal, cardiovascular and ocular systems. MFS is caused by mutations in the fibrillin-1 gene (*FBN1*), and mutations in *FBN1 *are known to be responsible for over 90% of all MFS cases. However, locus heterogeneity was reported in the early 1990's, when a second locus 3p24.2-p25 was suggested to cause MFS [1]. This association was further confirmed when mutations were identified in the transforming growth factor ß receptor type II gene (*TGFBR2*), which maps to the corresponding chromosomal region, in patients with overlapping phenotypes of MFS and Loeys-Dietz syndrome (LDS1B; MIM#610168) [2-4]. Later, using a functional approach, mutations were identified in another receptor of TGF-ß receptor family, transforming growth factor ß receptor type I (*TGFBR1*) in association with MFS or related phenotypes LDS (LDS1A; MIM#609192) [3-5]. These and other findings, strongly suggested an important role played by TGF-ß receptors and TGF-ß signaling dysregulation in the pathogenesis of MFS and related phenotypes [6,7]. The TGF-ß signaling pathway regulates extracellular matrix formation through members of the TGF-ß superfamily and their receptors [8]. TGF-ß mainly functions by binding to three cell surface receptors, namely TGFBR1 (55 kD), TGFBR2 (80 kD) and transforming growth factor receptor type III (TGFBR3, 280 kD) [9]. TGFBR3 is the most abundantly expressed subtype, has high affinity for all three TGF-ß isoforms, and acts as an enhancer of the TGF-ß access to the other signaling receptors [10]. So far, no systematic search for TGFBR3 genetic variation associated with MFS and related phenotypes has been reported in the literature. To test the hypothesis that dysregulation of TGFBR3-associated TGF-ß signaling is implicated in MFS or related phenotype pathogenesis, we selected a cohort of 49 patients, fulfilling or nearly fulfilling the diagnostic criteria for MFS. The patients were known not to carry a mutation in the *FBN1 *gene (including three 5' upstream alternatively spliced exons), the *TGFBR1 *and *TGFBR2 *genes. Mutation screening for the *TGFBR3 *gene in these patients and in controls led to the identification of a total of ten exonic (one novel), four intronic (one novel) and a 3'UTR variant in the *TGFBR3 *gene. Our data suggest that variations in *TGFBR3 *gene appear not to be associated with MFS or related phenotype.

## Results and Discussion

In a cohort of 49 unrelated probands with the tentative diagnosis of Marfan syndrome or fulfilling criteria of the "revised Ghent nosology" of 1996 [11] without identified mutation in the *FBN1, TGFBR2*, and *TGFBR1 *coding regions, a systematic mutation screen was performed by sequencing all 17 exons of *TGFBR3 gene*. A total of ten exonic (one novel), four intronic (one novel) and a 3'UTR sequence alterations were detected. Molecular findings of all index patients and relatives carrying variants in *TGFBR3 *gene are summarized in table 1.

**Table 1:** Variants identified and their respective allele frequencies in the *TGFBR3 *gene

Variants	Location	Amino Acid	Allele freq. Patient (n = 49)	Allele freq. Controls	**Ref. Acc. Nr**.	**Allele freq**.
c.44C > T	Exon 2	S15F	0.11	0.12 (n = 54)	rs1805110	0.325

c.55A > G	Exon 2	T19A	0.02	0.00 (n = 54)	Novel	

c.62-51 C > T	Intron 2		0.01	0.03 (n = 52)	rs17881268	0.03

c.216G > A	Exon 3	A72A	0.32	0.35 (n = 52)	rs2810904	0.407

c.247-40C > T	Intron 3		0.18	0.13 (n = 45)	rs11165441	0.13

c.886-1 0A > G	Intron 7		0.01	0.00 (n = 40)	Novel	

c.1128C > T	Exon 9	I376I	0.01	0.00 (n = 55)	rs11466595	0.015

c.1206G > A	Exon 9	P402P	0.41	0.41 (n = 55)	rs1805112	0.477

c.1341C > T	Exon 9	S447S	0.02	0.02 (n = 55)	rs2229500	ND

c.1566 + 55C > A	Intron 10		0.26	0.28 (n = 58)	rs7524066	0.19

c.2028C > T	Exon 13	F676F	0.51	0.41 (n = 59)	rs1805113	0.417

c.2247C > T	Exon 14	T749T	0.07	0.07 (n = 50)	rs284878	0.196

c.2293G > C	Exon 15	G765R	0.02	0.00 (n = 50)	rs17882828	0.034

c.2329C > T	Exon 15	P777S	0.01	0.00 (n = 50)	rs35352606	0.01

c.*19G > A	3'UTR		0.13	0.25 (n = 52)	rs1131243	0.10

The numbering is based on the mRNA sequence (*TGFBR3*; accession number NM_003243.4), where 1 corresponds to the nucleotide A of ATG, the translation initiation codon.

ND; not yet determined.

Among the exonic variants identified; c.44C > T (p.S15F; exon 2), c.216G > A (p. A72A; exon3), c.1128 (p.I376I; exon 9), c.1206G > A (p.P402P; exon 9), c.1341C > T (p.S447S; exon 9), c.2028C > T (p.F676F; exon 13) and c.2247C > T (p.T749T; exon 14) were detected in index patients in the same allele frequency as controls. Bioinformatic analyses using the online-software Mutation Taster, PMut and PolyPhen2 did not assign any disease-causing effect to these variants. Two already known exonic variants c.2293G > C (p.G765R; exon 15) and c.2329C > T (p.P777S; exon 15) were only detected in two and one index cases respectively, but not in controls. The first index case with the c.2293G > C variant was a 17-year-old male, who fulfilled the Ghent major criterion in the skeletal system, showed the involvement of the cardiovascular system and had a negative family history. The second index case with the c.2293G > C variant was a male sporadic case with suspected MFS and he was 26 years of age at the time of examination. The skeletal system was involved (body proportions, positive thumb and wrist signs, scoliosis, highly arched palate, typical facial features) and a major criterion would have been fulfilled, if he had been tested positive for the presence of protusio acetabuli. He had mitral valve prolapse. The variant c.2293G > C was present in his mother, who had no signs of MFS, and was absent in the healthy father.

The variant c.2329C > T was identified in a 14-year-old boy with involvements of the skeletal system and the skin. He had normal height at the age of 12-years, a slight funnel chest, flat feet, positive thumb and wrist signs, highly arched palate and joint hypermobility with recurrent herniae. Further anomalies were hypodontia (aplasia of 9 teeth), dysmorphic ears and stenosis of the external auditory meatus. At the age of 13-years celiac disease was diagnosed. His parents did not have signs of MFS, his mother was hypodontic but we were unable to screen the mother for the presence of this variant. This variant was identified in the healthy father.

The online-program PMut predicted both variants, c.2293G > C and c.2329C > T to be possibly pathogenic. On the contrary, the online-software Mutation Taster and PolyPhen2 did not assign any disease-causing effect to these variants. Taken the analysis of family members into account, both variants are apparently not disease-causing.

In our cohort, we encountered three known and one novel intronic variant in the *TGFBR3 *gene. Three known intronic variants c.62-51C > T (intron 2), c.247-40C > T (intron 3) and c.1566 + 55C > A (intron 10) along with 3'UTR variant (c.*19G > A) occurred in the index cases in the same allele frequency as in control cases. However, intronic variant c.886-10A > G (intron 7) is novel and was identified in a MFS case, who was later confirmed to carry a *FBN1 *mutation. Bioinformatic analyses using the online-programs Mutation Taster, Fruitfly and NetGene2 Server did not assign any disease-causing effect to these variants.

The only novel exonic variant c.55A > G (p.T19A; exon 2) was identified in two index cases with positive family history. The first index case was a 34-year-old male with marfanoid habitus and aortic aneurysm. The affected maternal uncle of this index case who also had Marfanoid habitus and aortic aneurysm, was wild type for c.55A > G but carried another variant c.44C > T (p.S15F; exon 2). The mother of the index patient had a marfanoid habitus as the only symptom of MFS and did not carry c.55A > G. A healthy sister of the index case carried c.55A > G and the son of the deceased daughter of the maternal uncle did not carry c.55A > G.

**Figure 1:**
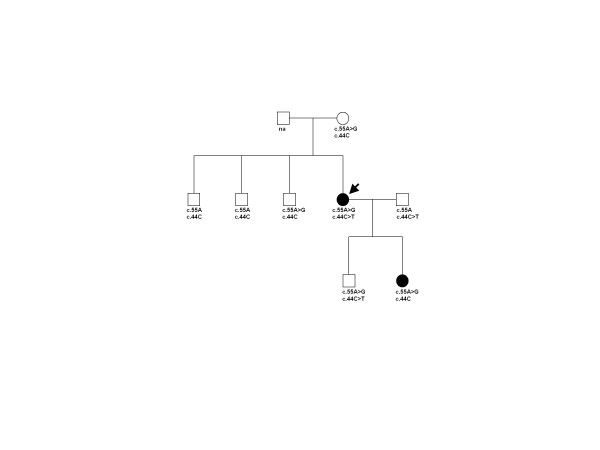
**Pedigree of a family with Marfan syndrome associated with c.44C > T (p.S15F) and c.55A > G (p.T19A) in the *TGFBR3 *gene**. The Index patient is indicated by arrow. na: no DNA available.

Another index patient with c.55A > G was a 40-year-old female with a mild dilatation of the aortic root (3.5 cm), when she was a young adult. As the dilatation was not progressive, the diameter of the aortic root was in the normal range when she got older. She had skeletal involvement (arm-span to height ratio >1.05, positive thumb and wrist signs, flat feet, highly arched palate) and had a history of two spontaneous pneumothoraxes. She had frequent nasal bleeding and easy bruising without trauma or varicosis. The affected daughter, who carried variant c.55A > G was 8 years of age when examined and had a dilatation of the aortic root with a diameter of 2.5 cm. Skeletal system was involved (positive thumb and wrist signs, flat feet and joint hypermobility). Additionally she had muscular hypotonia. A healthy son, brother and the mother of the index patient also carried c.55A > G. The healthy son of the index patient carried another variant c.44C > T as well. Two other healthy brothers and the husband did not carry the c.55A > G variant, but the husband carried the c.44C > T variant (figure 1). Both of these exonic variants occurred in a highly conserved TGFBR3 signal domain (table 2). A possible interpretation of c.55A > G; T19A is that it may be a predisposing factor to the aortic dilatation, as it affects a highly conserved signal domain (http://www.uniprot.org/uniprot/Q03167) and plausibly could affect the function of TGFBR3. c.55A > G; T19A may thus act as a mutation with reduced penetrance or perhaps as a variant that in combination with variation in other genes could lead to aortic dilatation. The bioinformatic prediction tool (http://www.cbs.dtu.dk/services/SignalP/) showed, however, that both c.55A > G sequences were predicted to be a valid signal sequences.

**Table 2:** Amino acid sequence comparison (44C > T; S15F and 55A > G; T19A) of the highly conserved TGFBR3 signal domain from *Homo sapiens *(accession no. NP_003234.2), P*an troglodytes *(accession no. XP_513555.2), *Sus scrofa *(accession no. NP_999437.1), *Mus musculus *(accession no. NP_035708) and *Rattus norvegicus *(accession no. NP_058952.1)

Species	Amino acid sequence
*H. sapiens*	**S**CLA**T**AGPEP
*P. troglodytes*	**S**CLA**T**AGPEP
*M. mulatta*	**S**CLA**T**AGPEP
*S. scrofa*	**S**CLA**T**AGPEP
*M. musculus*	ACLA**T**AGPEP
*R. norvegicus*	ACLA**T**AGPEP

Amino acid residues found to show variation as identified in this study are highlighted in bold and respective conserved amino acids are shown in bold and underlined.

## Conclusions

Taken together our data demonstrate that at least in our cohort, variations in *TGFBR3 *gene do not appear to play a role in the aetiology of MFS or related phenotypes, although the role of *TGFBR3 *variants as a genetic modifier can not be ruled out. Identification of known and novel variants in the current study could be useful in the studies of the other related disease aetiopathogeneses.

## Materials and Methods

### Probands

49 unrelated individuals used in this study had been referred between 1997 and 2005 to our clinic or genetic testing service with suspected Marfan syndrome or fulfilling Ghent diagnostic criteria of Marfan syndrome. These patients, had already been screened for 65 along with additionally three 5' alternatively spliced exons of *FBN1 *gene, 8 exons of *TGFBR2 *gene, and all 9 exons of *TGFBR1 *gene as described before [11-14] and were found not to carry a disease-causing mutation. Blood samples were taken and genomic DNA was extracted using standard protocols. Primers were designed based on the human sequence (accession number AY796304.1) for all 17 exons of *TGFBR3 *gene (table 3). To analyse the exonic variants we used the bioinformatic prediction programs Mutation Taster (http://www.mutationtaster.org/), PMut (http://mmb2.pcb.ub.es:8080/PMut/) and PolyPhen2 (http://genetics.bwh.harvard.edu/pph2/). All intronic variants and the variant in the 3'UTR were analysed with Mutation Taster (http://www.mutationtaster.org/), Berkeley Drosophila Genome Project "Splice Site Prediction" (http://www.fruitfly.org/seq_tools/splice.html) and NetGene2 Server (http://www.cbs.dtu.dk/services/NetGene2/). Patients carrying *TGFBR3 *variants were re-contacted in order to be checked for MFS, LDS related and/or additional symptoms.

**Table 3:** Sequences of primer pairs used for amplification of all 17 exons and the 3'UTR of *TGFBR3 *gene

Name	Primer sequences
**Exon 1F**	5'- AGG-GAG-GGC-GAG-TGC-GCC-GGG-T-3'
**Exon 1R**	5'- GGA-GGT-CCT-GGC-GGC-TGG-AGC-G-3'

**CDS 1F**	5'- GTC-TGT-GCT-CTG-AGC-AGC-CTG-AAG-3'
**CDS 1R**	5'- TCA-TCT-CAA-CTA-AAG-AGA-CTG-GGA-3'

**CDS 2F**	5'- GGC-ATC-TCT-GGT-GGG-TTG-GCA-GTG-3'
**CDS 2R**	5'- GCA-GAC-TCA-GTG-GCA-GTG-GGC-TGA-G-3'

**CDS 3F**	5'- GTA-TTC-CAG-AGG-CTG-CTC-TGA-G-3'
**CDS 3R**	5'- GAC-TCT-GGC-ATT-ATT-TCA-GTG-AAA-G-3'

**CDS 4F**	5'- CTT-CGA-TTT-GAG-AAG-TAC-TTT-CTC-T-3'
**CDS 4R**	5'- AAC-AAT-TGC-CTG-TCA-TAA-ATC-AGT-C-3'

**CDS 5F**	5'- GAA-TCT-GGT-TAC-CGA-GTA-CCT-CAG-3'
**CDS 5R**	5'- TCT-CCC-TGC-CTC-AAG-TCA-AGG-AAG-3'

**CDS 6F**	5'- GAC-ACT-AGA-AAC-ATG-AAG-ACT-TGG-3'
**CDS 6R**	5'- GAG-CTT-AGA-GAG-TCC-AAA-GAG-GCA-G-3'

**CDS 7F**	5'- CTA-AAG-TAC-TGT-TTA-ATT-TTA-GA-3'
**CDS 7R**	5'- CAT-ATA-AGC-TGA-AAT-GAC-AGT-TCC-3'

**CDS 8F**	5'- GTG-GCC-TGG-CAT-CAA-ACA-CTG-CTG-3'
**CDS 8R**	5'- CAG-ATG-CAG-ACT-AGG-GCC-AGA-TGG-3'

**CDS 9F**	5'- GTG-TCA-ATT-ATA-CAA-CAG-AAC-TGC-3'
**CDS 9R**	5'- CCC-TCT-TCA-TCT-TCA-AAG-AAA-TGT-T-3'

**CDS 10F**	5'- GAA-CCA-AAC-ACA-CAT-GGT-TTG-GTG-3'
**CDS 10R**	5'- GAT-AGT-CCC-TAA-CTA-AAG-CCA-ACA-A-3'

**CDS 11F**	5'- ATC-CTT-CAT-ATG-ACT-GTC-ATT-AAT-C-3'
**CDS 11R**	5'- GTA-TTT-TAG-CTG-ATG-TCT-AAG-GAA-C-3'

**CDS 12F**	5'- CCT-AAA-GTG-AAA-GTG-AGA-TGC-TAA-C-3'
**CDS 12R**	5'- CCT-CAC-CTA-AAA-ATG-CCA-AAA-TAA-C-3'

**CDS 13F**	5'- GTA-GAG-CTG-GTG-AAG-GCA-CTT-TTG-3'
**CDS 13R**	5'- GGT-CTT-CTT-AAC-AAG-CAG-AGC-TCA-G-3'

**CDS 14F**	5'- ATC-ATT-GAC-AGA-GCT-TTC-TCA-CAG-T-3'
**CDS 14R**	5'- GAA-TGA-GAG-CAG-AAG-TCT-CCT-TAT-C-3'

**CDS 15F**	5'- TGC-AAT-GCA-TGA-TGC-AGA-CTA-ACC-A-3'
**CDS 15R**	5'- ACA-AGC-TGT-TCA-CCA-ACT-CTT-ACT-C-3'

**CDS 16F**	5'- GGA-ATG-CAC-ATA-CAT-AAT-ATG-CGT-C-3'
**CDS 16R**	5'- GAA-TAC-AAC-GGG-TGA-TCT-TTA-TAC-3'

### PCR and DNA Sequencing

Standard PCR conditions were initial denaturation at 95°C for 10 min followed by 33 cycles of 96°C for 1 min, 55°C for 1 min and 72°C for 1 min with final elongation for 10 min at 72°C in a 50-μl reaction mixture, containing 1X buffer (Qiagen, Germany), 1X Q solution (Qiagen, Germany), 20 pM each primer and 2.5U Taq Polymerase (Qiagen, Germany). The annealing temperature for exon 1 and 15 were 65°C and 58°C, respectively. PCR products were purified with ExoSAP-IT (USB, USA), and both strands were sequenced with BigDye Terminator chemistry version 1.1 by standard protocol (ABI, USA). Sequencing reactions were carried out at 96°C for 10s, 50°C for 5s, and 60°C for 4 mins (25 cycles) (Biometra, Germany). The reaction mixtures were purified using DyeEx™ 2.0 Spin Kit (Qiagen, Germany) and analyzed on the ABI Genetic Analyser 3100 according to the supplier's instructions with the sequence analysis software (ABI, USA).

### Controls

All sequence alterations were checked in a sample of 55 healthy control blood donors.

## Competing interests

The authors declare that they have no competing interests.

## Authors' contributions

KKS carried out the molecular genetic studies, performed the sequence alignment and drafted the manuscript. BK participated in the sequence alignment. KKS, JS and MA-K conceived of the study, and participated in its design and coordination. All authors read and approved the final manuscript.

## References

[B1] Collod G Babron MC Jondeau G Coulon M Weissenbach J Dubourg O Bourdarias JP Bonaiti-Pellie C Junien C Boileau C A second locus for Marfan syndrome maps to chromosome 3p24.2-p25 Nat Genet 1994 8 3 264 268 10.1038/ng1194-264 7632217 PMC2045693

[B2] Mizuguchi T Collod-Beroud G Akiyama T Abifadel M Harada N Morisaki T Allard D Varret M Claustres M Morisaki H Heterozygous TGFBR2 mutations in Marfan syndrome Nat Genet 2004 36 8 855 860 10.1038/ng1392 15235604 PMC2230615

[B3] Singh KK Rommel K Mishra A Karck M Haverich A Schmidtke J Arslan-Kirchner M TGFBR1 and TGFBR2 mutations in patients with features of Marfan syndrome and Loeys-Dietz syndrome Hum Mutat 2006 27 8 770 777 10.1002/humu.20354 16799921

[B4] Loeys BL Chen J Neptune ER Judge DP Podowski M Holm T Meyers J Leitch CC Katsanis N Sharifi N A syndrome of altered cardiovascular, craniofacial, neurocognitive and skeletal development caused by mutations in TGFBR1 or TGFBR2 Nat Genet 2005 37 3 275 281 10.1038/ng1511 15731757

[B5] Loeys BL Schwarze U Holm T Callewaert BL Thomas GH Pannu H De Backer JF Oswald GL Symoens S Manouvrier S Aneurysm syndromes caused by mutations in the TGF-beta receptor N Engl J Med 2006 355 8 788 798 10.1056/NEJMoa055695 16928994

[B6] Neptune ER Frischmeyer PA Arking DE Myers L Bunton TE Gayraud B Ramirez F Sakai LY Dietz HC Dysregulation of TGF-beta activation contributes to pathogenesis in Marfan syndrome Nat Genet 2003 33 3 407 411 10.1038/ng1116 12598898

[B7] Byers PH Determination of the molecular basis of Marfan syndrome: a growth industry J Clin Invest 2004 114 2 161 163 15254580 10.1172/JCI22399 PMC449756

[B8] Pepin MC Beauchemin M Collins C Plamondon J O'Connor-McCourt MD Mutagenesis analysis of the membrane-proximal ligand binding site of the TGF-beta receptor type III extracellular domain FEBS Lett 1995 377 3 368 372 10.1016/0014-5793(95)01378-4 8549757

[B9] Cheifetz S Bassols A Stanley K Ohta M Greenberger J Massague J Heterodimeric transforming growth factor beta. Biological properties and interaction with three types of cell surface receptors J Biol Chem 1988 263 22 10783 10789 2899081

[B10] Lopez-Casillas F Wrana JL Massague J Betaglycan presents ligand to the TGF beta signaling receptor Cell 1993 73 7 1435 1444 10.1016/0092-8674(93)90368-Z 8391934

[B11] De Paepe A Devereux RB Dietz HC Hennekam RC Pyeritz RE Revised diagnostic criteria for the Marfan syndrome Am J Med Genet 1996 62 4 417 426 10.1002/(SICI)1096-8628(19960424)62:4<417::AID-AJMG15>3.0.CO;2-R 8723076

[B12] Rommel K Karck M Haverich A von Kodolitsch Y Rybczynski M Muller G Singh KK Schmidtke J Arslan-Kirchner M Identification of 29 novel and nine recurrent fibrillin-1 (FBN1) mutations and genotype-phenotype correlations in 76 patients with Marfan syndrome Hum Mutat 2005 26 6 529 539 10.1002/humu.20239 16220557

[B13] Rommel K Karck M Haverich A Schmidtke J Arslan-Kirchner M Mutation screening of the fibrillin-1 (FBN1) gene in 76 unrelated patients with Marfan syndrome or Marfanoid features leads to the identification of 11 novel and three previously reported mutations Hum Mutat 2002 20 5 406 407 12402346 10.1002/humu.9075

[B14] Singh KK Shukla PC Rommel K Schmidtke J Arslan-Kirchner M Sequence variations in the 5' upstream regions of the FBN1 gene associated with Marfan syndrome Eur J Hum Genet 2006 14 7 876 879 10.1038/sj.ejhg.5201620 16617303

